# The Impact of Health and Education on Labor Force Participation in Aging Societies: Projections for the United States and Germany from Dynamic Microsimulations

**DOI:** 10.1007/s11113-023-09781-3

**Published:** 2023-04-26

**Authors:** René Böheim, Thomas Horvath, Thomas Leoni, Martin Spielauer

**Affiliations:** 1 Johannes Kepler University Linz and Austrian Institute of Economic Research (WIFO), Keplergebäude 117A, Altenbergerstraße 69, 4040 Linz, Austria; 2 Present Address: Austrian Institute of Economic Research (WIFO), Arsenal Objekt 20, 1030 Vienna, Austria; 3 University of Applied Sciences Wiener Neustadt, City Campus, Schlögelgasse 22-26, 2700 Wiener Neustadt, Austria

**Keywords:** Employment projections, Dynamic microsimulation, What-if-scenarios, Health, C5, J11, J21

## Abstract

We project the labor force in the United States to 2060 and contrast the outcomes with comparative projections for Germany. In both countries, the population will age, but the demographic dynamics are fundamentally different. According to our dynamic microsimulations, the labor force in the U.S. will increase by 17 percent between 2020 and 2060 (about 29 million workers) despite population aging. In contrast, the labor force in Germany will decline by 11 percent (about 4.5 million workers). Our baseline projections indicate that an expansion of education will increase the labor force by about 3 million persons in the United States and about half a million persons in Germany by 2060. In several what-if scenarios, we examine the effects of further expanding education and of removing health barriers on labor force participation. Higher educational attainment among those with currently low education has the largest impact on labor force participation, relative to the additional years of schooling. However, health improvements and the labor market integration of people with health limitations suggest a larger increase in labor force participation rates. Using Sweden as a benchmark, we show that reducing the health participation gap would increase the U.S. labor force by as much as 13 million people in 2060 (+6.8 percent compared to our baseline).

## Introduction

Demographic aging is a major challenge for future economic growth and the sustainability of public finances. Across the OECD, the ratio of people aged 65 and over to people of working age (15 to 64) is projected to rise from 25 older persons for 100 working-age persons in 2018 to 40 older persons for 100 working-age persons in 2050 (OECD, 2019). For a proper assessment of the economic implications of aging, it is, however, essential to go beyond purely demographic considerations, as rising labor market participation rates (LFPRs) may compensate for a smaller working-age population. Projections of labor force participation are crucial for assessing future revenues and costs of the social security system.

We develop a dynamic, multi-country microsimulation model to project age-specific labor force participation in the United States and draw international comparisons while accounting for education and health. There is a long tradition of microsimulation modeling in the U.S., going back to models such as the DYNASIM model developed by the Urban Institute in the 1970s (Favreault et al., 2015). The microsimulation model that we use is highly stylized, focusing on the major determinants of labor force participation, but with the advantage of a high level of transparency and international comparability and, ultimately, the ability to contrast different what-if scenarios.

We use the model to compare labor force developments in the United States with developments in Germany and contrast different policy scenarios. On the aggregate level, both the starting population and its evolution are modeled to be consistent with the demographic structures and projections provided by the Census Bureau for the United States and Eurostat for Germany. We compare the U.S. with Germany because Germany is the largest European economy and has numerous features typical of European welfare states, such as (almost) universal public healthcare and an overall strong social safety net. The comparison is of interest also because the two countries have had different long-term demographic and labor force participation trends in the recent past. Demographic aging is more advanced in Germany and, in contrast to the U.S., the labor force is expected to shrink in absolute terms in the coming decades. At the same time, Germany introduced several structural reforms to its labor market and pension system (Seeleib-Kaiser, 2016) and – unlike the United States – has experienced a continuous rise in labor force participation rates.

To assess how education and health reforms might affect labor force participation in the coming decades, we select a series of what-if scenarios. We first examine the implications of improvements in the educational structure of the population. Secondly, we use scenarios that highlight the potential effects of health improvements and policy changes that improve the labor market integration of working-age persons with health limitations. The OECD considers the large number of people who leave the labor market due to health limitations or disability a “social and economic tragedy” (OECD, 2010, p. 9). Many countries have been reforming their social security provisions over the last two decades to improve the prevention and management of health-related work incapacity (Böheim & Leoni, 2018). Future labor market participation will also depend on developments in population health. While life expectancy is projected to increase in the coming decades, it is less clear what impact this will have on the number of healthy life years and thus working life expectancy.

## Background

### Projecting Labor Force Participation

Projections of labor force participation are a key component of the study of the effects of aging. Generally speaking, two types of modeling approaches are used in this field: macro-models and microsimulation. In macro-models, labor force projections typically take the form of cohort models. The Congressional Budget Office, for instance, uses a cohort model that estimates labor force participation rates by age-sex-education and race/ethnicity subgroups (Montes, 2018). The model used by the Social Security Administration Office of the Chief Actuary (2019) projects the civilian labor force by age, sex, marital status, and presence of children. The U.S. Bureau of Labor Statistics (BLS) applies participation rate projections by age, gender, race and ethnic groups, which are developed using data from the Current Population Survey (CPS, 2019), to population projections produced by the Census Bureau (BLS, 2021a). In Europe, the discussion of economic and social policy issues is informed by the macroeconomic scenarios included in the E.U. Commission’s Ageing Report (European Commission, 2017, 2018, 2020, 2021). In the Ageing Report projections, employment rates are extrapolated into the future using a dynamic cohort model based on age-dependent probabilities of labor market entry and exit over the previous ten years.

These approaches place high demands on the consistency between assumptions of different modeling levels. Microsimulation provides a more integrated approach for consistently modeling downstream effects, distinguishing composition effects from behavioral effects, and – in general – the incorporation of theory and policy in projections. For example, Spielauer and Dupriez (2019) use microsimulation for modeling inter-generational educational dynamics and the analysis of downstream effects of education policy interventions on fertility, child immunization, stunting, and infant mortality in developing countries.

Microsimulation has proven to be a particularly useful tool to answer ‘What if…?’ questions (Zaidi & Rake, 2001). Van Hook et al. (2020), for instance, use a dynamic microsimulation model to evaluate the long-run implications of various immigration policy proposals for the skill levels of the future labor force in the United States. Their demographic model (LSD–USA) was originally developed for Canada by the Laboratoire de Simulation Démographique (LSD-C) and has been used, *inter alia*, to study the influence of literacy skills on the Canadian labor force (Vézina & Bélanger, 2020). Other models of this family were adapted for European countries (Marois & Aktas, 2021; Marois et al., 2019a, 2019b, 2020).

### Education and Health as Determinants of Participation

**Fig. 1:**
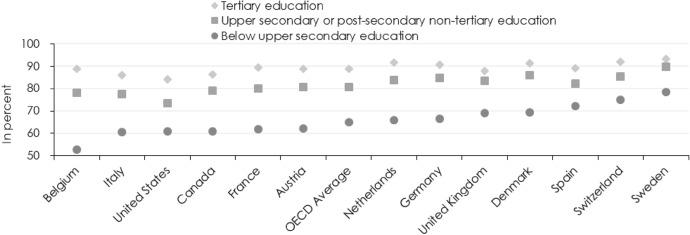
Labor force participation rate, age 25 to 64, by educational level. Source: OECD.Stat (2020) [Educational attainment and labor-force status]. – Educational level according to ISCED 2011 A

Across the OECD, on average, the labor force participation rate of individuals who completed tertiary education is about 24 percentage points higher than the rate for individuals who have not completed high school, i.e., an upper secondary education (Fig. 1). In both the United States (23.3 percentage points) and Germany (23.9 percentage points), this difference is close to the OECD average.

In virtually all countries, the educational participation gap is much more pronounced for women than for men (OECD.Stat, 2020). The gap amounts to 33.2 percentage points for women and 15.8 percentage points for men in the United States; in Germany, the difference is 29.4 percentage points for women and 16.4 percentage points for men. The labor supply of women is influenced by several factors associated with gender roles and aspirations (Del Boca, 2002; Fernández et al., 2005; Folbre, 1994; Jaumotte, 2003). These factors interact with education and educational choices, resulting in a stronger positive correlation between educational attainment and labor force participation for women than for men. Formal education and future educational trends can thus be expected to impact the projection of labor force participation rates more strongly for women than for men.

**Fig. 2:**
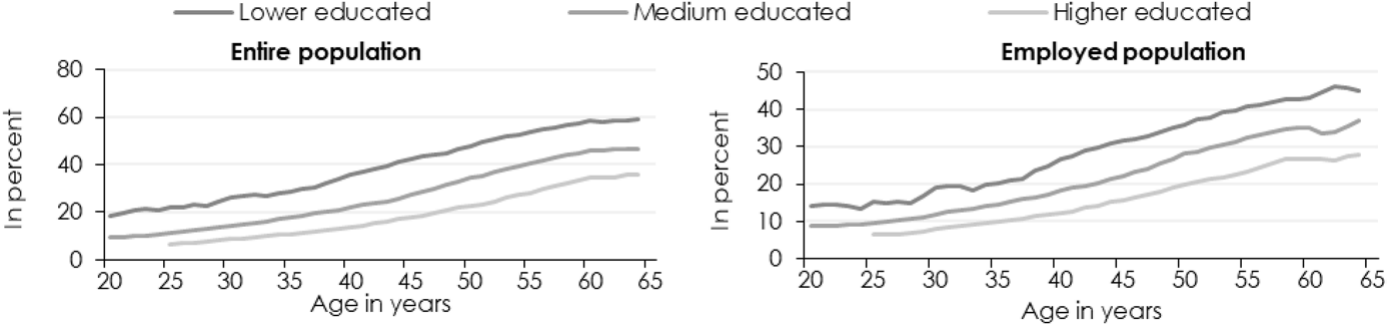
Share of people reporting poor health by employment status and education, OECD countries, age 20 to 64. Source: OECD (2017). – Based on pooled waves of different survey microdata. Health status is self-reported. Poor health refers to the answer categories lower than “good”, i. e. “very bad”, “bad” and “fair” (as opposed to “good” and “very good”) or “poor” and “fair” (as opposed to “good”, “very good” and “excellent”), depending on the survey

Like education, health correlates positively with labor force participation, and health and education are also positively correlated with each other (Grossman, 2015; Lundborg, 2013). Figure 2 shows that the correlation between education and health is stable over the life-cycle, and it can be observed for both the employed and the total population. However, measuring the causal links between education and health is challenging, not least because of third factors that may cause health and education to vary in the same direction (Cutler & Lleras-Muney, 2010; Eide & Showalter, 2011; Grossman, 2015). In our microsimulations, we will model health depending on demographic characteristics as well as education.

Future labor market participation will thus depend, at least in part, on the health of the population. Life expectancy is projected to increase in the coming decades, even in the United States, where a decline was observed in the second half of the 2010s (Board of Trustees, 2020; Vespa et al., 2020).^1^ However, it is less clear to what extent the number of healthy life years and thus working life expectancy will change. Increases in life expectancy might be accompanied by an increasing prevalence of morbidity and disability, as medical advances prevent an increasing number of fatal outcomes but more people live with disabilities and chronic conditions (“expansion of morbidity”). Alternatively, disease prevention and behavioral changes could result in later onsets of disease and disability, thus prolonging healthy life years and increasing working life expectancy (“compression of morbidity”). Next to these two hypotheses, which have been formulated by Gruenberg (1977) and Fries (1980), respectively, there is a third, somewhat intermediate, position proposed by Manton (1982). According to this “dynamic equilibrium” hypothesis, delays in the progression from less severe to more severe disabilities will result in an increase in moderate disability prevalence, but a decrease in severe disability prevalence in the population. The question which of these hypotheses fits best is primarily an empirical one and might be answered differently depending on the country and time period studied (Crimmins & Beltrán-Sánchez, 2011; Lee et al, 2020).

Poor health and disability are generally associated with reduced chances of obtaining and remaining in employment (Baumberg Geiger et al., 2019; Schuring et al., 2007). The extent to which health impacts labor market activity does, however, vary greatly in international comparison, and the effect also depends on labor market institutions and policies. Disability pensions are an area where this international heterogeneity is particularly evident (Börsch-Supan et al., 2009). The labor market inclusion of workers with health problems does however not depend only on the design of disability benefits but on a broader range of policies to promote employment and support reintegration (OECD, 2010).

### Policies for the Inclusion of Workers with Health Problems

Many countries have been reforming their social security provisions to improve the prevention and management of health-related work incapacity. Overall there was a broad shift towards policies that support the labor market activation of people with health problems (Böheim & Leoni, 2018; OECD, 2003, 2010). Typical measures are, among others, the introduction of early intervention programs, the promotion of vocational rehabilitation measures, a stronger focus on workers’ residual work ability, as well as the involvement of employers in preventing a permanent exit from the labor market in case of illness.

Despite a common trend towards increasing activation, sickness and disability policies still vary substantially between countries. In the United States, there were few reforms of sickness and disability during the last two decades and policies to support the labor market integration of people with health limitations are underdeveloped. Morris (2016) points out that the lack of reform activity can be explained, at least partly, with the structural arrangements of the Social Security Disability Insurance (SSDI). Baumberg Geiger et al. (2019) stress that the U.S. is exceptional as it is the only country of the 12 they compared where the employment of older workers with ill-health deteriorated between 2004 and 2015.

In contrast, Germany took several steps to strengthen the integration dimension of its sickness and disability policies, notably with the institutionalization of disability management (*Betriebliches Eingliederungsmanagement*) (Bernhard et al., 2011). Other policies were the introduction of the right to rehabilitation (in 2001) and of sickness absence monitoring (2004). The reform of 2004 also requires firms to offer employees who are sick for more than 6 weeks support for their return to work. More recently, Germany undertook steps to increase the use of graded sick leave schemes, aimed at facilitating return-to-work after longer health-related absences from work (Leoni, 2022).

Countries that have dealt more intensively with integration policies have higher activity rates or have at least experienced a more favorable development over time than those which lacked these reforms. Switzerland, Sweden, and the Netherlands, for example, achieved a marked decline in disability benefit claims and an increase in employment rates of older workers with poor health (Hemmings & Prinz, 2020; Koning & Lindeboom, 2015; OECD, 2014).

### Demographics and Labor Force Participation in the United States and Germany

The United States and Germany differ in their demographic and labor force participation dynamics. According to recent census projections (Medina et al., 2020), total life expectancy in the U.S. is projected to increase from 79.7 in 2017 to 85.6 years by 2060. Over the same period, the population is projected to grow by nearly 79 million people, from about 326 million to 404 million (Vespa et al., 2020). The size of the older population is projected to grow even faster, and by 2060 at least 25 percent of the U.S. population is projected to be at least 65 years old. An increase in the labor force participation rate could help to cushion the negative impact of aging. However, labor force participation has not evolved favorably in the United States in the recent past. After peaking around the year 2000 at about 67.3 percent (age 16 and older), the U.S. LFPR declined until about 2015 and has remained stable at about 63 percent since then (Perez-Arce & Prados, 2021). During the same period, the LFPR of most other economically advanced countries increased (OECD, 2021). Aaronson et al. (2014) and Krueger (2017) suggest that about half of the decline in the U.S. is due to long-running demographic changes. In their summary of the literature, Perez-Arce and Prados (2021) argue that this could even amount to two-thirds.

However, other factors, such as increased schooling, the opioid epidemic, and social security programs such as the Social Security Disability Insurance (SSDI) may have also contributed to the decline in the LFPR. A detailed analysis by demographic sub-groups reveals a reduction in participation for prime working-aged persons and the youth of both genders (Abraham & Kearney, 2020). Between 2000 and 2017, the participation rate of young men dropped from 68.1 percent to 56.3 percent, and for young women from 63.2 percent to 54.1 percent. The participation rate of prime working-age men declined slightly in this period, from 91.5 percent to 89.1 percent. At the same time, the LFPR of prime working-age women, which had risen to 77 percent in the late 1990s and remained stable there until around 2000, fell to 75.2 percent in 2017. Overall, the BLS expects the LFPR for the total population to decline, from 63.1 percent in 2019 to 61.2 percent by 2029 (Dubina et al., 2020). In its 2021 long-term budget outlook, the CBO assumes that the LFPR for the total population (aged 16 or older) will decline to 60.2 percent in 2060 (CBO, 2021).

Unlike the U.S., where the population is set to keep growing, the demographic projections by the European Commission (2021) indicate a declining population size for Germany between 2019 and 2060, from about 83.1 million to about 81.8 million people. The share of the population over 65 years of age is projected to grow from 21.7 percent in 2019 to 28.3 percent by 2060. At the same time, the share of the population aged 20 to 64 years is projected to decline from about 60 percent in 2019 to about 52 percent in 2060.

The LFPR of the German working-age population has increased substantially between 2000 and 2019.^2^ According to the Ageing Report, labor force participation rates are projected to remain constant between 2019 and 2060 for younger persons (aged 20 to 24) and to increase slightly for those aged 25 to 64 years. The LFPR of old persons (65–74) is projected to increase from 13.9 percent in 2019 to 18.3 percent in 2060, reflecting legislated pension reforms. In the aggregate and because of changes in the demographic composition of the workforce, the LFPR of the population aged 20 to 74 years is estimated to decline slightly, from 73.1 in 2019 to 71.9 percent in 2060.

## Data and Modeling Approach

### General Approach

We develop the dynamic microsimulation model “microWELT-US”. In Europe and the U.S., dynamic microsimulation is predominantly used in pension simulation models (Gál et al., 2009). Such models are usually very complex country-specific applications based on detailed national data. Our modeling approach takes a different path, trading complexity for transparency and—based on internationally available data—supporting the comparative study of the impact of policies in different countries.

Multi-country comparative applications are a recent development in microsimulation. The benefits are currently demonstrated primarily in static tax-benefit microsimulations, as with the European Union tax-benefit microsimulation model Euromod (Browne & Immervoll, 2017; Sutherland & Figari, 2013). An example of a dynamic multi-country model which, among other applications, was also used for labor force projections is the collection of LSD-Models (Van Hook et al., 2020; Vézina & Bélanger, 2020; similarities and differences between LSD and microWELT-US are discussed below). MicroWELT-US builds on the microWELT modeling platform, and recent work extends this platform for labor force projections in Europe (Horvath et al., 2021). microWELT was designed as a versatile, extendable, and portable tool for comparative studies of welfare transfers and is extensively documented in Spielauer et al., (2020a, 2020b), Amann et al. (2021), and the project website microWELT.eu.

The core of microWELT-US consists of demographic models, supplemented with socio-economic processes (education and employment) and the modeling of health statuses. By simulating individuals in their family context, intergenerational processes such as the intergenerational transmission of education are considered. microWELT-US explicitly models mortality, fertility, the formation and dissolution of partnerships, partner matching, education, migration, leaving parental home, health, and labor force participation. Education influences the labor force directly and indirectly via differences in family characteristics (e.g., lower fertility of higher educated women), the education-specific prevalence of health limitations, and differential mortality. One of the key features of microWELT-US is the inbuilt ability to reproduce existing population projections in aggregate outcomes such as age-specific fertility, mortality by age and sex, and net migration by age and sex. Our only demographic assumptions refer to differences by education in fertility (age of first childbirth, childlessness) and mortality, which we assume to remain constant over time. The differences in demographic trends that we observe in the projections for the U.S. and Germany are largely due to differences in fertility and not because of different net migration rates, which are very similar for both countries (0.30% of the population per year in the U.S. vs 0.29% in Germany).

MicroWELT-US and LSD share the same programming technology as both are implemented in Modgen, a generic microsimulation language developed and maintained at Statistics Canada. Like LSD, microWELT-US combines a continuous-time, competing-risk approach for its demographic core modules with cross-sectional imputation models used for the periodic (monthly) update of labor force participation. However, there also exist some critical differences in the respective approaches. Unlike LSD, microWELT is a time-based interacting population model allowing the communication between simulated persons and introducing a central observer keeping track, communicating, and reacting to aggregate simulation outcomes at any moment during the simulation. The time-based design – each simulated unit passing through time simultaneously, rather than being simulated one by one – is a prerequisite for some of the central model features of the microWELT platform, including the modeling of partnerships and family links, the intergenerational transmission of education, and model alignment to aggregate targets.

### Data

MicroWELT is a comparative model and its parameters are entirely based on publicly available standardized data. For the U.S., we use the Annual Social and Economic Supplement (ASEC) of the CPS public use file data (2017). For Germany, data from the 2014 European Union Statistics on Income and Living Conditions (EU-SILC) and the 2017 EU-SILC ad-hoc module “Health and children’s health” are used, containing health-related variables not included in the standard EU-SILC data. To model the intergenerational transmission of education, we use the information on respondents’ and their parents’ educational attainment in the OECD PIAAC data for the U.S. and the 2009 ad-hoc module “Entry of young people into the labor market” of the EU-Labor Force Survey (LFS).

The starting populations of the simulations are constructed from cross-sectional data of the same sources (ASEC; EU-SILC) that represent the underlying populations in 2014 for Germany and 2017 for the United States. The simulation size (the number of simulated persons) is independent of the size of the starting population and can be freely chosen. The model also allows several replicates to be simulated in parallel to provide distributional information on random fluctuations in the results (Monte Carlo Variation). Results presented in this paper are based on 12 replicates of initial 167.000 persons each, i.e., 2 million.

Population projections and their underlying assumptions concerning fertility, mortality, and net migration are taken from the U.S. Census Bureau and Eurostat.

### Modeling Labor Force Participation, Education, and Health

Labor force participation is modeled using logistic regressions based on age, sex, education, health status, and—in the case of women – the presence of dependent children and the age of the youngest child. The estimations are based on 2017 CPS data for the U.S. and EU-SILC data for Germany. They are performed separately by sex and age group: persons under 25 years of age (also accounting for education enrolment), persons of prime working age (25 to 54), and, finally, persons of higher working age (55 and older). The labor force participation of women of prime working age is estimated separately by the presence of dependent children. Tables  5 and 6 in the Appendix report odds-ratios from the logistic regressions for the U.S. and Germany. In general, our estimations take the form.

1 $${\text{ln}}\left( {\frac{p}{1 - p}} \right) = \beta_{0} + \beta_{e1} x_{e1} + \beta_{e2} x_{e2} + \beta_{e3} x_{e3} + \beta_{h} x_{h} + \mathop \sum \limits_{i = 1}^{{\text{m}}} \beta_{ai} x_{ai} + e,$$

where *p* is the probability of labor force participation. The parameters to be estimated are (*ß*_*0*_,..) and are estimated by maximum likelihood. ${x}_{e1}$ to ${x}_{e3}$ are binary variables which indicate the highest level of education according to our classification of education (with base category 0 corresponding to the lowest level of education, *e*_*1*_ denotes high-school graduation, *e*_*2*_ indicates some college, and *e*_*3*_ indicates a university degree). ${x}_{h}$ is a binary health indicator where the value 1 indicates impaired health and 0 no health problems, ${x}_{a}$ is a set of *m* age-group binary indicators (5-year age-groups for young and prime age workers and single year age indicators for the retirement age group). For prime working age mothers, additional controls ${x}_{y}$ for the age of the youngest child are added in the form of 4 age-group binary indicators which indicate the age of the youngest child in the family in one of the age categories.

For prime-age mothers our estimation takes the form

2 $$\ln \left( {\frac{p}{1 - p}} \right) = \beta_{0} + \beta_{e1} x_{e1} + \beta_{e2} x_{e2} + \beta_{e3} x_{e3} + \beta_{h} x_{h} + \mathop \sum \limits_{i = 1}^{{\text{m}}} \beta_{ai} x_{ai} + \mathop \sum \limits_{j}^{4} \beta_{yj} x_{yj} + e.$$

Adding an indicator for current education participation ${x}_{edu}$ and neglecting the health indicator (due to the very small number of persons with health-impairment at younger ages) for the youngest age group (age 15 to 24) results in an estimating equation of:

3 $${\text{ln}}\left( {\frac{p}{1 - p}} \right) = \beta_{0} + \beta_{e1} x_{e1} + \beta_{e2} x_{e2} + \beta_{e3} x_{e3} + \beta_{edu} x_{edu} + \mathop \sum \limits_{i = 1}^{{\text{m}}} \beta_{ai} x_{ai} + e.$$

Based on the coefficients obtained from these logistic regressions, we calculate individual probabilities of labor force participation.^3^ Each individual is then assigned their labor force participation status randomly by drawing a random number between 0 and 1. If this number exceeds the estimated probability, labor force status is set to zero, and one other wise. We update the labor force status monthly for each individual based on the estimation results from these regressions. As individuals age over time and possibly change their education or health status, their labor force participation probability is updated throughout the simulation.

For the oldest age group, changes in retirement age legislation are incorporated when determining labor force participation. We achieve this by shifting the age parameter from our logistic regression models backwards as retirement age increases: If, for example, retirement age increases by one year from 65 to 66, the likelihood of labor force participation of a 60-year-old person is determined by using the estimated coefficient for age 59 instead of age 60. If retirement age increases to age 67, the estimated coefficient for age 58 is used to determine the probability of a 60-year-old person, and so on. Thus, with retirement age 67 our model implies that a 60-year-old person has the same labor force participation rate as a 58-year-old person with identical characteristics (gender, education, and health status) when retirement age is 65. By explicitly taking health status into account, the effect of raising the retirement age on labor force participation is thus dampened. In this way, we account for the fact that the effect of increases in retirement age on labor force participation is non-linear since sickness-related withdrawals from the labor force partly counteract such an increase.

For the United States, we assume – in line with current legislation – that the retirement age at full-benefits gradually increases to 67 for people born in 1960 or later. Early retirement benefits will still be available at age 62 (although the benefits will be reduced over time). For Germany, we also follow current legislation and assume a one-time increase in the regular retirement age to 67 years in 2030, up from 65.8 years in 2020, and a constant early retirement age at 63 years.

Formal educational attainment is classified into four levels. Acknowledging the difference between the German and the U.S. educational system, we use slightly modified classifications for the two countries. For Germany, the four levels correspond to the ISCED levels 0–2, 3, 4, and 5 + . ISCED 4 is of high importance in Germany (apprenticeship after graduating from high-school) but has no proper correspondence in the education system of the U.S. In our classification, in the U.S. the four levels refer to (1) “below high-school” (up to grade 9), (2) “high-school” (grade 10–12), (3) “(some) college”, and level (4) “university”.

The modeling of education attainment combines information from the starting population with trend scenarios and scenarios based on the intergenerational transmission of education, i.e., the influence of the parents’ education on their children’s education. The latter is the main mechanism chosen in our – rather conservative – base scenario in which changes in the education of parents entirely drive future education trends.

We model three education transitions. Concerning the first transition (high school graduation), we assume that for persons who are at least 22 at the start of the simulation (i.e., those born before 1996), an observed education level “below high school” is final, and no further education transition takes place. High school graduation is still possible for those between 16 and 21 (those born between 1996 and 2001), and their graduation probability follows a logistic (flattening) trend estimated over the past two decades. For birth cohorts from 2002 onwards (younger than 16 at the start of the simulation), high school graduations are entirely determined by the intergenerational transmission of education. Since virtually all persons aged 16 and younger still live with (at least one of) their parents, we use the parents’ education to model the probability of high school graduation. These probabilities are based on odds ratios for the transition rates from one education level to the next are reported in Table 9 in the Appendix.

Higher education transitions (attaining some college or a university degree) are modeled following the same logic. Again, for the 2002 and younger cohorts, all education transitions are determined by intergenerational transmission. However, those born after 1991 (age 25 or younger at the start of the simulation) who graduated from high school can still attain “some college” or a university degree. The same applies to those born before 1987 (30 or younger) who at least have “some college” and may still achieve a university degree.

Figure 13 in the Appendix depicts the education composition of the prime working population aged 25 to 59 from 2020 to 2060. According to our baseline projections, the two highest education groups in both countries increase in size. However, the United States currently has a significantly higher population share with (at least some) college education. In contrast, Germany is undergoing a more substantial educational expansion. The influence of parents’ education is considerably stronger in the United States compared to Germany, and intergenerational mobility is thus higher in Germany.

**Fig. 3:**
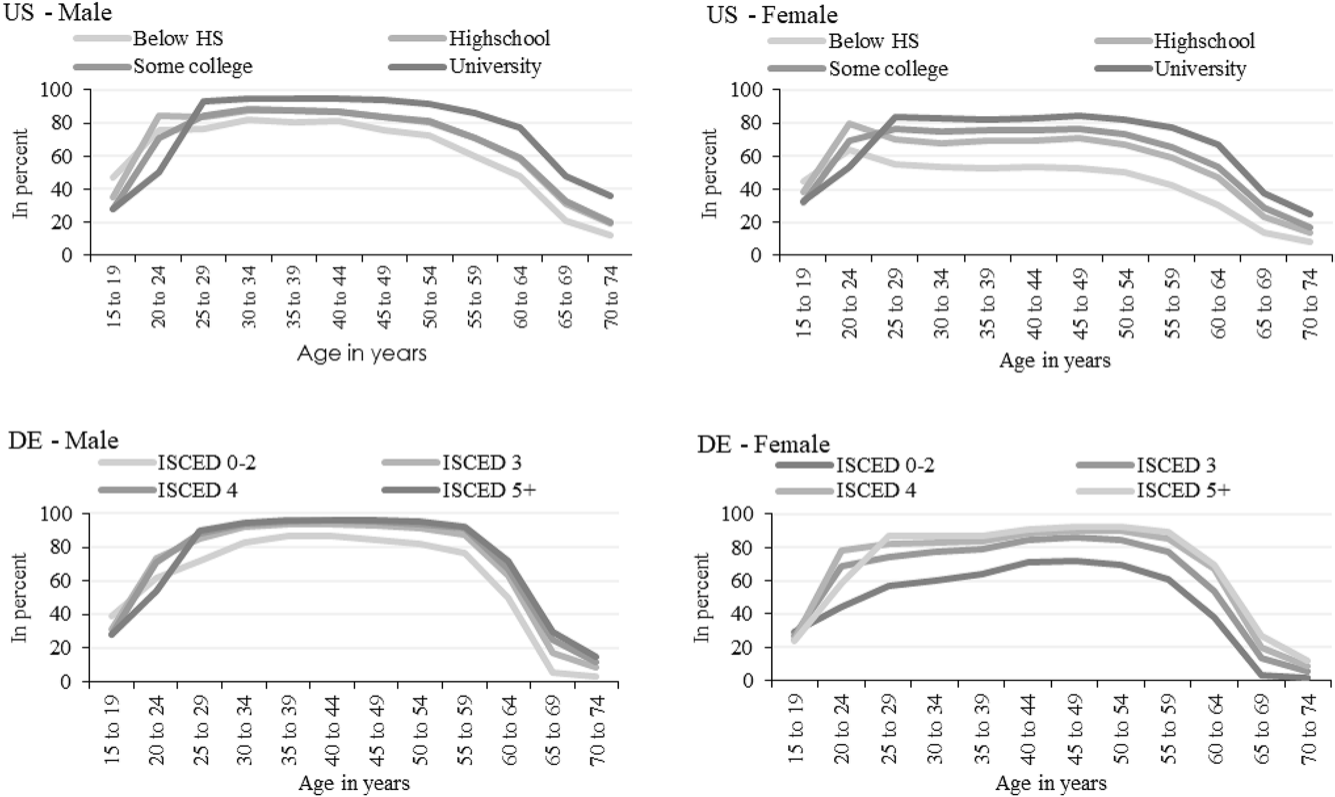
Labor force participation by age and highest level of education. Simulated data for 2020 based on calculations using ASEC (2017) and EU-SILC (2014) data

We observe a positive education gradient in labor force participation (Fig. 3). In accordance with the literature, the variation in simulated participation by education is more pronounced for women than for men. This is particularly evident when comparing the two lowest education groups with the remaining groups.

Comparing the U.S. to Germany, we find the most notable difference for the two highest education levels. In the U.S., participation rates of persons with (some) college and completed university education differ significantly. In contrast, in Germany, both men and women with ISCED 4 level have a participation behavior that is similar to that of the group with university education. In Germany, the ISCED 4 level mainly includes persons who complete apprenticeship training after high school. About 20 percent of a cohort has both a high-school leaving certificate and completed an apprenticeship (Bellmann & Prümer, 2021). This indicates the strong labor-market orientation of the dual education track that characterizes the German educational system.

Health status is modeled with a latent health indicator that allows individuals within each country to be ranked by health. Rather than attempting to define an absolute health measure and to compare persons with the same health status across countries, we thus focus on relative differences in health status within each country. This choice is motivated by limitations in the comparability of available health measures. The ASEC and EU-SILC datasets do not contain the same survey items. More importantly, there is abundant evidence indicating that different types of people have different ways of reporting their health across different times and places, even when standardized questionnaires are used (Angelini et al., 2011; Jürges, 2007; Kalwij & Vermeulen, 2008). This difficulty to interpret self-reported health measures becomes apparent when we look at the large variation in the number of persons reporting functional limitations. For instance, according to EU-SILC data for 2017, 19.2 percent of the German population aged 20 to 69 years reported limitations in activities because of health problems, while the corresponding share was 29.3 percent in Austria and 6.9 percent in Sweden.

We follow an approach developed by Poterba et al. (2013) and combine different variables into a single measure of latent health using a principal component analysis (PCA). For the United States, we use the following health indicators of the 2017 CPS ASEC data: self-rated health (scaled from poor to excellent), difficulty in doing daily activities (binary), and presence of a health problem or a disability which prevents work or which limits the kind or amount of work (binary). For Germany, we use EU-SILC 2017 data with the ad-hoc module “Health and children’s health” that provide a broader set of health measure. In addition to self-rated health, we use the following indicators: presence of a chronic illness (binary), limitation in activities that people usually do (no limitation vs. limited vs. strongly limited), body-mass-index (in four categories from underweight to very obese), as well as the number of visits to the general practitioner and number of visits to medical specialists in the last 12 months.

The health index is generated using the first principal component from the PCA of these variables, which represents the weighted average of the health indicators (where the weights are chosen to maximize the proportion of the variance of the individual health indicators that can be explained by the first principal component). The first principal component can be interpreted as a latent health index. We use this to construct percentile scores of each individual’s position in the health distribution in a country. In the projection model, poor health rates are kept constant by age, gender, and level of education.

**Fig. 4:**
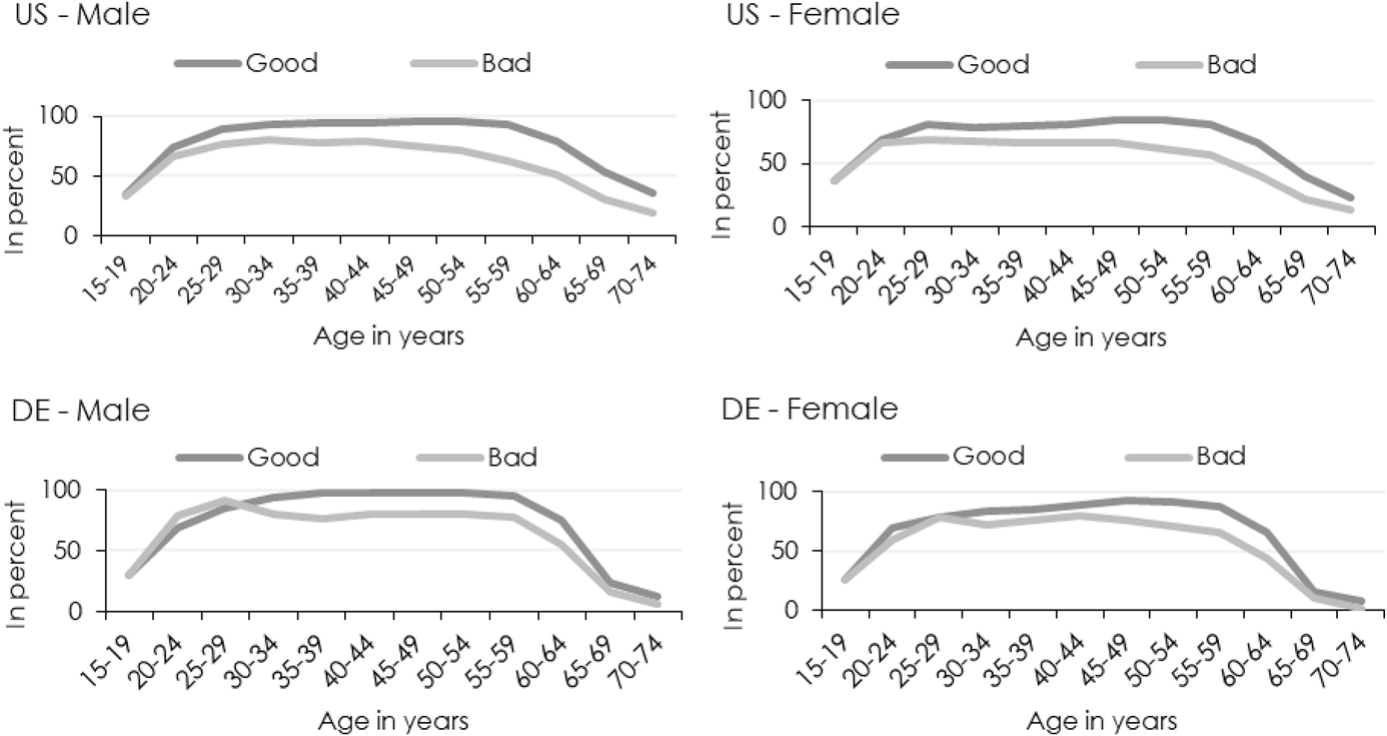
Participation rates for individuals in good and poor health, by age. Simulated data for 2020 based on calculations using ASEC (2017) and EU-SILC (2014; 2017) data. People in bad health are defined as those in the bottom tertile of the health distribution, as measured with a latent health indicator based on several health indicatorsincluded in the surveys

This approach aims to identify, for each country separately, equally sized groups of people with relatively poor health and compare their labor market outcomes with those of persons with better health. Persons whose health indicator is in the lower third of the distribution are considered to have health restrictions. The indicator increases with age and is negatively correlated with education. Both in the United States and Germany, the participation rates of persons who report poor health are below those of persons in good health for men and women of all age groups (Fig. 4).

The major caveat associated with this approach is that people who are in the same position of the health distribution in their respective country might actually have different health statuses in absolute terms. As we can see in Fig. 14, however, the health gradient in participation rates is remarkably similar in the United States and Germany (with low participation in the first tertile of the distribution). The only exception is the population at the very bottom of the health distribution, which is less likely to participate in the labor market in the United States than in Germany. This deviation might point to differences in the health status of people at the bottom of the health distribution in the two countries. Several studies report that adults in the U.S. have worse health than adults in Europe and other high-income countries and that the difference is particularly pronounced among socio-economically disadvantaged groups (f.i. Avendano et al., 2009; Woolf & Aron, 2013).

### Policy Scenarios

To assess how reforms addressing education and health might affect labor force participation in the coming decades, we select a series of what-if scenarios. We first examine the implications of improvements in the educational structure of the population. Secondly, we use scenarios that address the impact of health on labor force participation.

### Education

For the first set of scenarios of educational reforms, we choose scenarios with pronounced shifts between educational levels. These affect, by assumption, 25 percent of the selected educational groups. Note that the proportion of the cohort affected by these shifts depends on the initial size of the affected education group. For example, if only 10 percent of a cohort belong to the lowest education group, a policy that successfully moves 25 percent of those to the next higher attainment level would affect only 2.5 percent of the cohort. In our scenarios S1–S3 of higher transitions between two adjacent educational levels, we do not change the proportions of people at the remaining (higher) levels. For example, when we assume that a policy moves 25 percent of persons from the first to the second education level (i.e., from “below high school” to “high school”), this only increases the size of the second education group and leaves the higher education groups unaffected. These scenarios are particularly helpful in gauging and comparing the effects of specific forms of educational expansion.

The fourth scenario consists of a combination of the other three scenarios. It provides a benchmark for the effects we can expect due to a substantial improvement in future cohorts’ educational attainment. The resulting four scenarios are described in Table 1.

**Table 1:** Description of scenarios for increased educational attainment

Education scenario	Description of educational shift	Affected share of the respective education group (birth cohorts from 2010 onwards)	Affected share of the total 2010 birth cohort	
			United States	Germany
S1	From level 1 (below high school/ISCED 0–2) to level 2 (high school/ISCED 3)	25%	1.6%	2.2%
S2	From level 2 (high school/ISCED 3) to level 3 (some college/ISCED 4)	25%	5.6%	6.8%
S3	From level 3 (some college/ISCED 4) to level 4 (university/ ISCED 5 +)	25%	6.4%	4.1%
S4	From each educational level to the next-higher one	25%	All of the above combined	

A caveat of our education scenarios concerns the relationship between a specific level of education and labor force participation, which might change over time. In our model, educational expansion is associated with a higher propensity to participate in the labor market. However, labor supply may depend on relative skill levels as well as on absolute skill levels as workers adapt their participation behavior to their labor market perspectives relative to the rest of the labor force. The relationship between education and participation in the cross section may overstate the extent to which increases in the level of education will raise participation. On a similar note, the “race between education and technology” (Goldin & Katz, 2009) may reduce the future demand for labor for a given education level. Recent studies on technological progress stress the negative effect of technological progress on labor force participation, especially of men (e.g., Autor & Dorn, 2013). In our microsimulations, we need to hold technologcal progress at current levels, which leads to a perhaps too optimistic development of labor force participation resulting from educational expansion. However, Grigoli, Koczan, and Toplova (2020) show that the negative effects of automation on labor force participation of prime-age persons are mitigated by higher spending on education and active labor market programs.

#### Health

The what-if scenarios of the second type of reforms highlight the potential effect of health improvements and policy changes that improve the labor market integration of working-age persons with health limitations. The two scenarios are described in Table 2. The first health scenario (S5) addresses the question of how the health status of the working-age population will develop over time. The baseline demographic projections consider changes in life expectancy over time, but they do not account for possible improvements in terms of healthy life years. Although there is uncertainty regarding future developments and the extent to which we will experience a “compression” or “expansion” of morbidity, evidence suggests that healthy life years and working life expectancy have been increasing (Weber & Loichinger, 2020). In our baseline scenario, the negative impact of demographic aging on the health composition of the workforce is thus likely overstated. In scenario (S5), we assume that increases in life expectancy lead to a proportional extension of healthy life years in the working-age population. For instance, if the life expectancy increases by five years, the group of those aged 60 to 64 years is attributed the same health structure that was previously displayed by those aged 55 to 59 years.

**Table 2:** Description of health scenarios

Health scenario	Description of health improvement
S5	Increase in life expectancy leads to a proportional extension of healthy life years in the working-age population
S6	Differences in labor market outcomes by health status in the U.S. and Germany converge to Swedish levels by 2060

In the second health-related scenario (S6), we highlight the potential of policies to improve the labor market inclusion of individuals with poor health. Table 3 compares the labor force participation rates of workers with poor health in the U.S., Germany, and three benchmark countries, using the CPS and EU-SILC data on which our dynamic microsimulation modeling is based. We select the Netherlands, Switzerland, and Sweden as benchmark countries because of their intense reform activities concerning sickness and disability policies. Workers with poor health are defined as those in the bottom tertile of the health distribution. The participation rates of workers with health problems, as well as the differential between workers in good or poor health, vary considerably. The United States have the lowest participation rates and the largest health-related participation gaps, with the exception of the age group 65 to 69, where the share of labor market participants is higher in the U.S. than in the other countries. While the Netherlands have similar values to Germany, Switzerland, and, in particular, Sweden have higher activity rates and smaller health-related gaps.

**Table 3:** Participation rates of persons with poor health, United States and Germany compared to selected countries

Age in years	Women					Men				
	US	DE	CH	NL	SE	US	DE	CH	NL	SE
Labor force participation rates of those in poor health (in %)										
50 to 54	61.5	73.0	82.6	60.4	84.9	71.6	81.4	89.9	71.9	87.5
55 to 59	56.1	70.4	70.0	58.7	84.5	62.6	80.0	84.1	67.2	90.0
60 to 64	41.4	55.3	56.2	40.6	69.5	50.8	57.6	68.0	52.6	69.1
65 to 69	21.3	1.9	5.4	8.2	13.9	30.6	1.8	22.4	19.9	16.8
Difference between good and poor health (in percentage points)										
50 to 54	22.9	15.0	− 1.8	17.8	5.2	23.7	13.0	4.2	17.2	6.3
55 to 59	24.6	11.9	5.5	13.1	3.9	30.5	9.4	6.9	20.3	2.9
60 to 64	24.4	8.3	2.1	7.7	5.2	28.5	12.8	6.5	17.6	10.2
65 to 69	19.0	1.3	4.4	0.9	1.6	22.8	2.7	− 0.3	− 1.7	4.4

We use Sweden as a benchmark in scenario S6 and assume that up to 2060, the impact of impaired health on labor market participation in the U.S. and Germany converges to observed differences by health status in Sweden of 2020. Because of differences in population health, achieving a substantial reduction in the health-related participation gap might be more difficult in the U.S. than in other countries. Workers in the bottom of the health distribution in the U.S. are likely to have on average a poorer health status than those in the bottom of the distribution in Sweden (Avendano et al., 2009; Woolf and Aron, 2013). While better policies could contribute to more of those in poor health remaining in work, it may not be possible to achieve results in the U.S. as good as those in the Scandinavian country.^4^ Our scenario S6 has therefore to be interpreted as an upper bound, indicating the scope for the expansion of labor force participation through both better policies and improved population health. Avendano and Kawachi (2014) argue that much of the U.S. health disadvantage is due to crucial differences in public policy, and especially in social policy, across the life course.

## Results

### Baseline

**Fig. 5:**
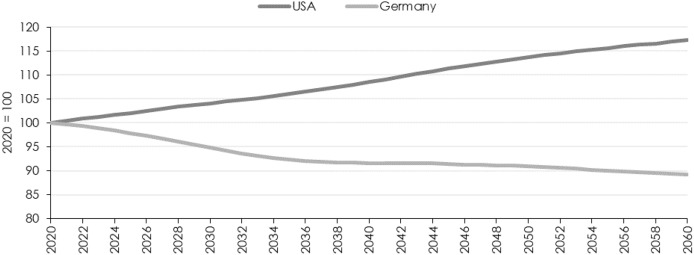
Change in the size of the labor force (relative to 2020)

Figure 5 shows the baseline simulations of the two countries’ labor forces relative to 2020. According to our dynamic microsimulations, the labor force in the U.S. will increase by 17.2 percent (corresponding to 28.8 million workers) between 2020 and 2060, while it will decrease by 10.7 percent (4.4 million workers) in Germany. The differences are largely due to the different population dynamics of the two countries, which are based on official population projections (Census Bureau for the U.S. and Eurostat for Germany): while the population in the USA will grow over the entire period under consideration, it will stagnate in Germany.

**Fig. 6:**
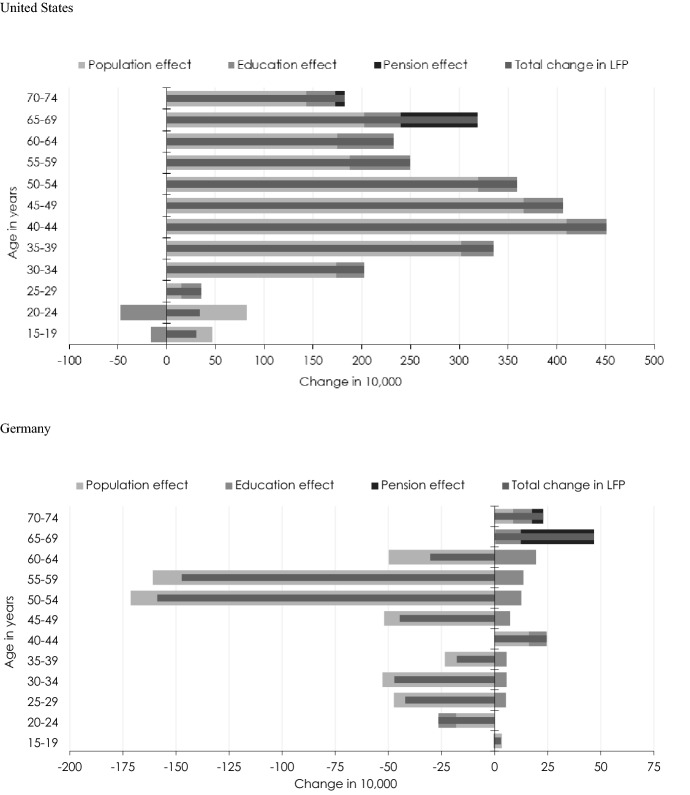
Decomposition of changes in the labor force between 2020 and 2060

Figure 6 shows the decomposition of the projected changes in the labor force into effects resulting from demographic change, changes in education, and pension reforms. In the United States, population growth increases the labor force in all age groups. In contrast, demographic change in Germany reduces the labor force in virtually all age groups, with a particularly large negative effect in the 50–59 age group. The U.S. labor force is projected to increase by about 24 million persons by 2060 due to demographic change (i.e. under the assumption that the age-specific labor force participation rates remain the same over time), while the labor force in Germany will decrease by almost 5.5 million people.

Apart from younger age groups, where more education leads to later labor market entry and thus reduces labor force participation at a young age (lock-in effect), more education increases labor force participation. We project that the educational expansion increases the labor force by about 3 million persons in the U.S. by 2060 and about half a million persons in Germany (compared to a scenario with constant education by sex and age group). In both countries, the largest effects are projected for the 55–64 age groups. This positive education effect increases when we extend the projection horizon, as the impact of additional education on labor force participation is strongest towards the end of the working career.

The projected effects from pension reforms are limited to older age groups, and are always positive. The baseline results project the U.S. labor force to grow by about 0.8 million persons between 2020 and 2060 and by about 0.4 million persons in Germany.

**Fig. 7:**
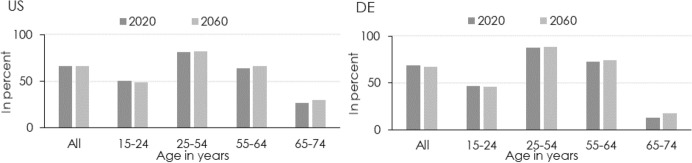
Labor force participation rates 2020 and 2060, baseline

Overall, we project the LFPR of 15- to 74-year-olds in the U.S. to be nearly the same in 2060 as in 2020 (67.0 percent) (Fig. 7 and Tables 7 and 8 in the Appendix). This is slightly higher than the most recent BLS and CBO projections, which project a slightly lower LFPR for 2060 (CBO, 2021; Toossi, 2016; BLS 2021b). Unlike the BLS and CBO projections, we do not differentiate by race/ethnicity, which could result in increased labor force participation.^5^For Germany, our projections show labor force participation rates that are, in the aggregate and up to the year 2030, similar to those in the E.U. Commission’s Ageing Report and are, after 2030, marginally higher (European Commission, 2018). The Ageing Report projects constant or slightly increasing participation rates of the younger age group (15 to 24 years). Our baseline results, in contrast, project a decline in labor force participation in the younger age group. This is consistent with the modeled educational expansion, as longer periods of education lead to delayed labor market entry and reduce labor force participation at younger ages.

### Scenarios

#### Education

##### Results from the Perspective of a Single Cohort (Year of Birth 2010)

**Fig. 8:**
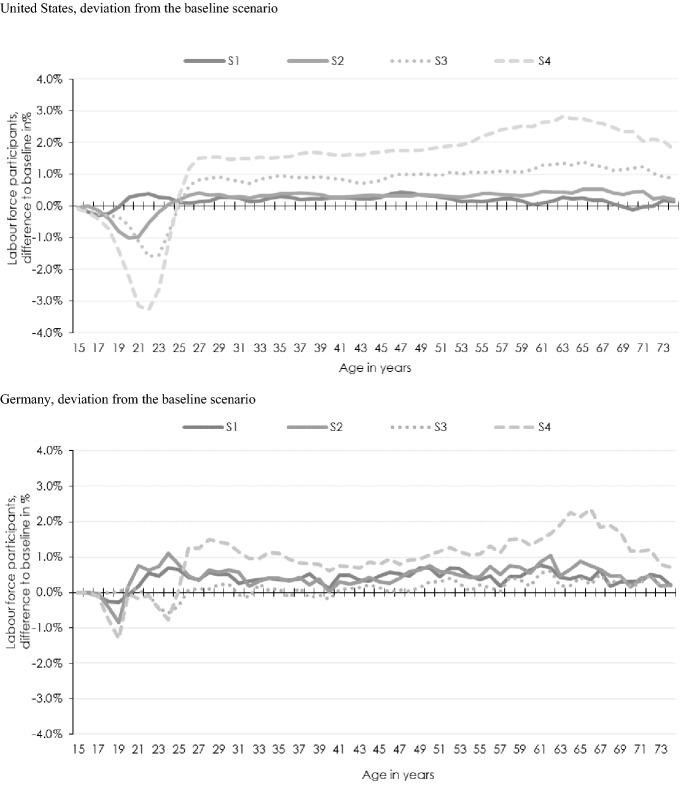
Change in the labor force of the 2010 birth cohort, by education scenario. Scenarios S1 to S4, as described in Table 1. Moving averages over three single year age groups

Figure 8 shows how the labor force participation of the cohort of 2010 changes over the life-cycle in the four different scenarios compared to our baseline scenario. The shift to higher educational attainment reduces labor force participation at younger ages due to increasing education phases. A positive effect on of labor force participation emerges between the age of 20 and 27, depending on the type of educational expansion implied by the scenario. Overall, the quantitative impacts of these educational expansions are moderate. The German cohort consists of about 750,000 persons and, depending on age, we observe increases in the labor force of up to 18,000 persons, i.e., roughly 2.5 percent of the cohort. The magnitude is similar in the U.S., where, starting from a birth cohort with about 4.1 million persons, we observe the maximum in scenario S4 with a projection of about 115,000 additional persons in the labor force at the age of 65 (corresponding to an increase of 3.0 percent).

Table 4 displays the cumulative effects of the scenarios for the 2010 birth cohort over the entire working life. To highlight that the effects are more pronounced towards the end of the working life, where labor force participation is presently below-average, we show the effects separately for ages 15 to 74 and ages 60 to 74. In the first scenario (S1), the number of person-years in the labor force increases by 0.5 percent in Germany (corresponding to an increase of about 176,000 person-years) and 0.2 percent in the U.S. (corresponding to about 420,000 person-years). The effects of the second scenario (S2) are slightly stronger, but otherwise similar in both countries. (It is important to note that scenario S2 affects a much larger share of the cohort than scenario S1.) The third scenario (S3) leads to a more modest effect in Germany than in the United States. This is because of the small difference in participation rates between high school graduates with a dual education compared to university graduates in Germany. In the U.S., on the other hand, university graduates are more attached to the labor market than persons with (some) college education. The absolute effects of scenarios S2 and S3 in the United States and S2 in Germany are larger than those of scenario S1, but S2 and S3 involve much higher shares of the population than S1 (see Table 1).

**Table 4:** Cumulative changes in labor force participation, by education scenario (S1 to S4)

Scenario	Labor force participation over the life course							
	US				DE			
	Age 15 to 74		Age 60 to 74		Age 15 to 74		Age 60 to 74	
	Years	In %	Years	In %	Years	In %	Years	In %
United States and Germany, deviation from the baseline scenario								
S1	416,718	0.2	55,343	0.3	175,616	0.5	49,490	1.0
S2	539,903	0.3	182,079	0.9	198,035	0.5	61,397	1.2
S3	1,682,042	0.9	553,673	2.7	55,206	0.1	35,733	0.7
S4	3,246,413	1.7	1,141,333	5.5	417,734	1.1	177,016	3.6

Scenarios S1 to S4, as described in Table 1. Years are person-years.

In the fourth scenario (S4), the labor force increases cumulatively by 418,000 person-years (1.1 percent) in Germany and 3.2 million person-years (1.7 percent) in the United States. In almost all scenarios, the most substantial effects are measured for the older age groups.

**Fig. 9:**
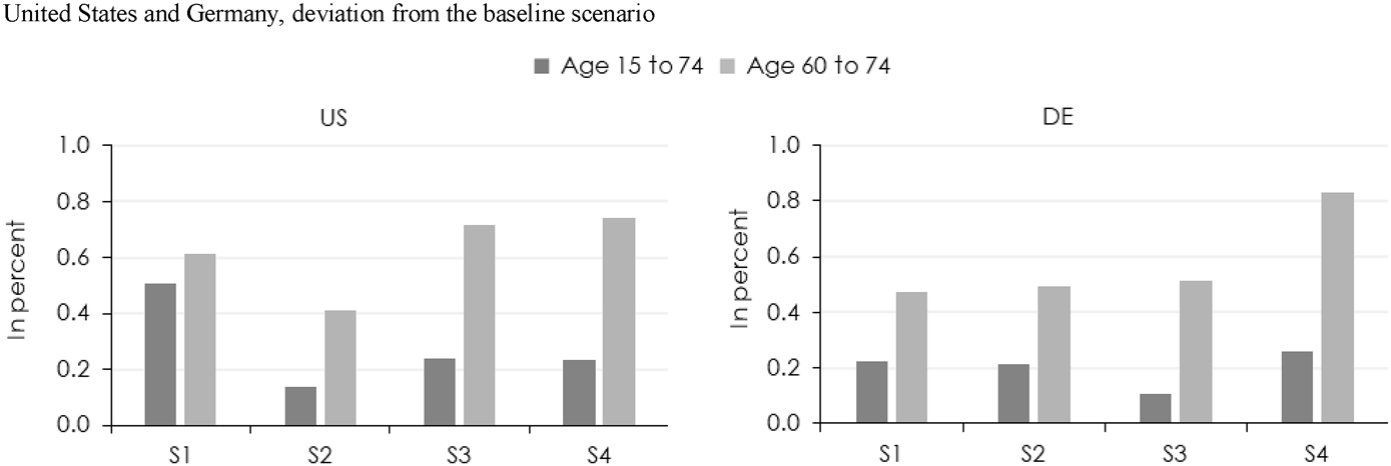
Standardized labor market effects, by education scenario (S1 to S4). Scenarios S1 to S4, as described in Table 1

Since the four scenarios imply transitions between educational levels of differently-sized population groups, Fig. 9 provides an overview of the standardized effects. The standardized effects are obtained by dividing the total changes in labor force participation displayed in Table 4 by the additional years of schooling that result from the respective scenario. The effects can be interpreted analogously to an elasticity, expressing the relative change in labor market outcome (labor force participation in years) for a 1-year-change in the number of years of schooling in the population (above age 15). Both the years-of-schooling and the labor market participation are measured cumulatively over the projection period.

In all scenarios, and in both countries, the relative effect of additional schooling is stronger towards the end of the working life than the effect on the entire working life (age 15 to 74). The first scenario, which increases the number of persons who attain high school education, has the strongest relative effects on participation over all age groups. For each additional year of schooling at the high school (ISCED 3) level, labor force participation increases by 0.51 percent in the United States and 0.21 percent in Germany. This comparatively large effect results from the fact that labor force participation rates among those with low education (ISCED 0–2 or “below high school”) are lowest among all education groups at most ages. Shifting people from intermediate to higher education levels results in more modest increases in labor force participation, as the differences in labor force participation rates are less pronounced compared to the lowest education level. (See also Fig. 3.)

##### Results from the Perspective of Target Years 2060 and 2080

**Fig. 10:**
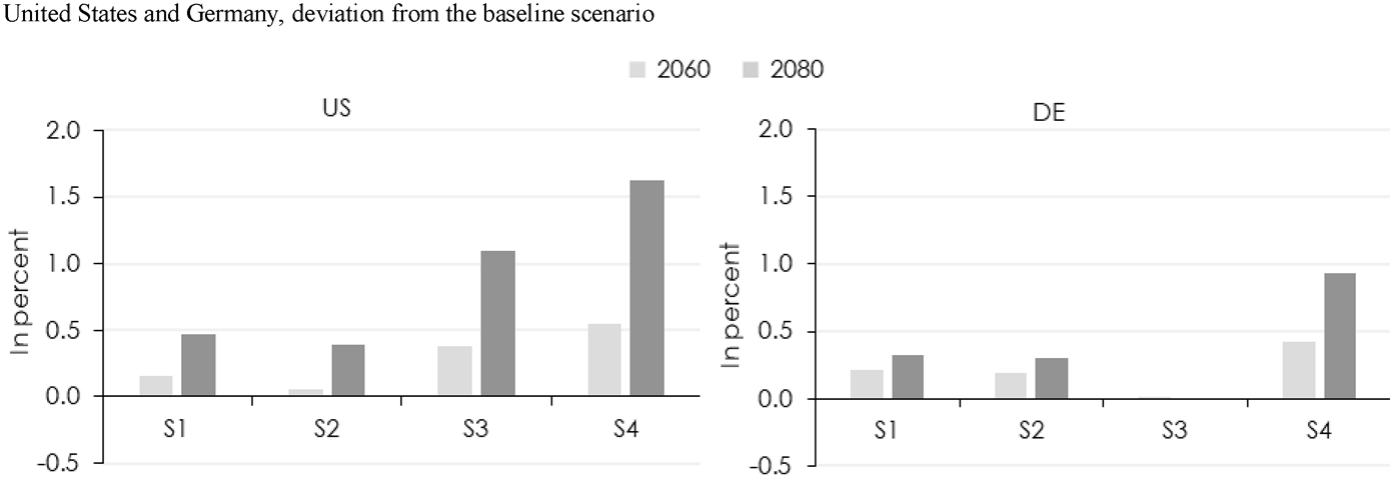
Total relative effects in 2060 and 2080, by education scenario (S1 to S4). Scenarios S1 to S4, as described in Table 1

This section presents the overall effects of the education scenarios (affecting all cohorts born 2010 and later) for individual target years. Concerning changes in education and their labor market effects, our main projection horizon until 2060 is comparatively short. In 2060, most of the cohort of 2010 will have spent fewer than 30 years in the labor market and will still have a part of their working life ahead of them. For this reason, Fig. 10 provides an overview of the results of the different scenarios, both for 2060 and 2080 (i.e. the year when the birth cohort 2010 will have turned 70 years of age).

The results show that a sizeable (further) educational expansion will have only a limited impact by 2060. The effects are stronger when we focus on the year 2080. We can expect a sizeable educational expansion (S4) to increase the German labor force by + 0.9 percent (approximately 350,000 persons) in 2080 compared to the baseline scenario. The lack of effects in scenario S3 for Germany is due to the small difference in participation rates between high school graduates with a dual education and university graduates. In the United States, scenario S4 corresponds to an increase in the labor force by 3.3 million workers in 2080, i.e. an increase of + 1.6 percent.

#### Health

**Fig. 11:**
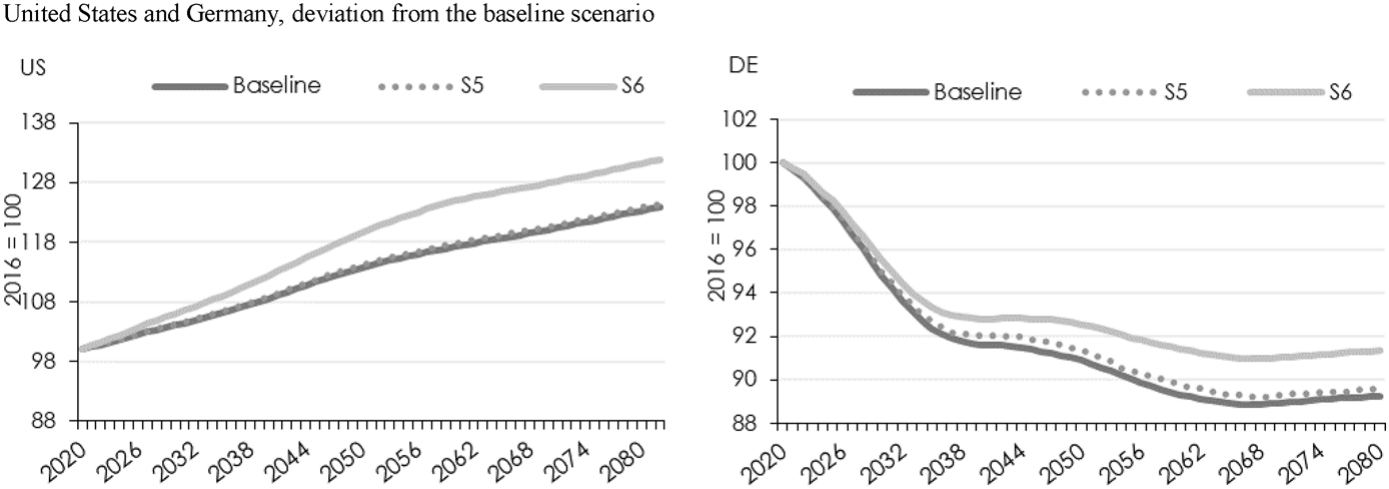
Change in the labor force, by health scenario. Scenarios S5 and S6, as described in Table 2

Figure 11 presents the results from the health-related scenarios S5 and S6, and compares them to the baseline.^6^ Scenario S5, where we assume a “slower aging” of the population and thus improvements in health status that mirror the extension of life expectancy, has only a small quantitative impact, which increases slightly over time. It should be stressed, however, that the assumption behind this scenario implies a sort of “dynamic equilibrium” in the development of life expectancy and healthy working life expectancy. This scenario is more optimistic than our baseline scenario, but it is cautious when compared with possible scenarios with more substantial improvements in population health.

**Fig. 12:**
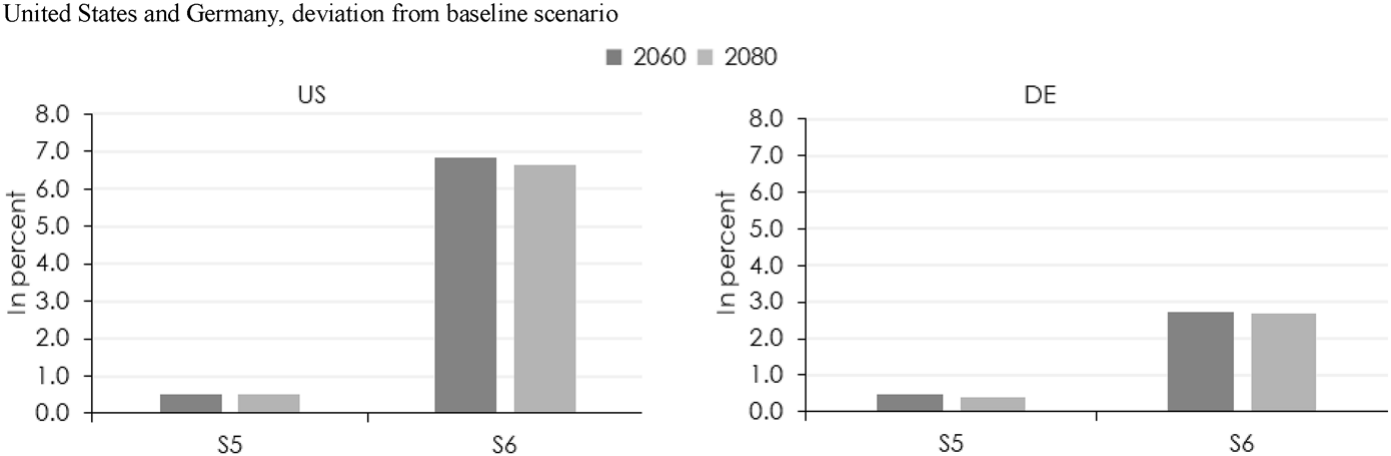
Relative effects in 2060 and 2080, health scenarios (S5 and S6). Scenarios S5 and S6, as described in Table 2

**Fig. 13:**
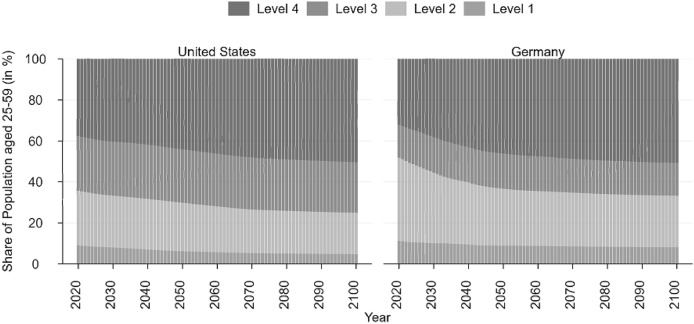
Education composition of the population 25–59 by calendar year. microWELT simulation results – base scenario. Own calculations based on CPS Annual Social and Economic Supplements 2017, OECD PIAAC data and EU-SILC 2014. Level 1: below high school (ISCED 2 for European countries); level 2: high school (ISCED 3); level 3: (some) college (ISCED 4); level 4: university (ISCED5 +)

**Fig. 14:**
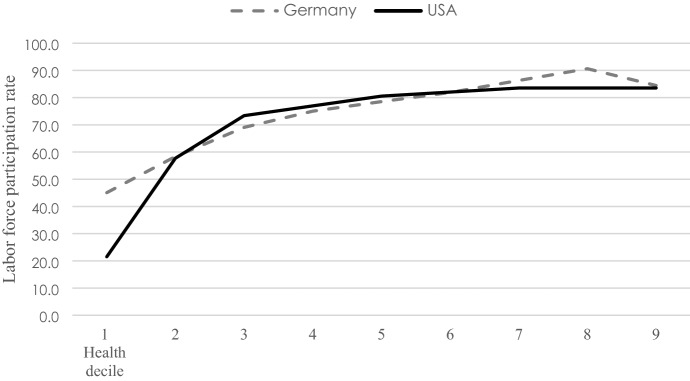
Labor force participation by health decile in the United States and Germany, age 15 to 74. Simulation based on ASEC (2017) and EU-SILC (2014; 2017) data

Scenario S6, on the other hand, with Sweden as the benchmark for the labor market integration of working-aged persons in the bottom tertile of the health distribution, shows that a reduction of the health gap in labor force participation can have a substantial impact on labor force dynamics. If Germany were to approach participation patterns currently displayed by Sweden, this would add about 1.05 million persons to the labor force by 2060 (+ 2.7 percent) and about 1.03 million by 2080 (+ 2.7 percent) compared to the baseline scenario described in Sect. 4.1, additionally compensating for around 17 to 21 percent of the decline implied by demographic aging (Fig. 12). The effects of reducing the health gap in participation rates would be even larger in the United States. Our microsimulation results indicate that scenario S6 would add 13.4 million persons to the workforce in 2060 (corresponding to + 6.8 percent compared to the baseline) and 13.6 million persons in 2080 (+ 6.6 percent). The effects are so strong, because currently health gaps in labor market outcomes are more pronounced in the U.S. than they are in Germany.

## Summary

We studied the effects of education and health reforms on the U.S. labor force with dynamic microsimulations until 2060 (with parts of the results up to 2080). The microsimulations explicitly model the determinants of labor force participation using a large number of simulated individuals in continuous time. They allow quantifying how changes in individual characteristics affect labor force participation through direct and indirect processes. Projected changes in labor force participation can be decomposed into underlying factors, such as population aging or changes in the population’s education structure. In our policy analyses, we focus on shifts in the educational structure and on improvements in population health and in the labor market integration of working-age persons with health limitations. We contrast the projected effects with projection results for Germany.

Both the U.S. and Germany are expected to undergo demographic aging, but their current demographic circumstances differ starkly. This has strong implications for their labor force developments. According to our microsimulations, the U.S. labor force will, despite population aging, increase by 17.2 percent in the age groups 15 to 74 (corresponding to 28.8 million workers) between 2020 and 2060, while Germany will experience a decline by 10.7 percent (4.4 million workers). In these baseline projections, improvements in the education structure will add about 2 million persons to the U.S. labor force and about half a million persons to the German labor force by 2060.

In a set of what-if-scenarios, we examine the implications of improvements in the educational structure of the population and of policies which address the health dimension of labor force participation. Of the what-if-scenarios that focus on education, relative to the number of additional school years, increasing the number of persons who achieve high-school education has the strongest positive impact on labor force participation. Since the number of people with less than high school education is comparatively small, however, the absolute effect on labor force participation from improving education only for this group is modest. Shifting people from intermediate to higher education levels increases the labor force participation in higher age groups, but this increase is partially offset by lock-in effects at younger ages, as higher education implies postponed labor market entry reducing labor market participation at younger ages. In almost all education scenarios, the most substantial effects are measured at the end of working careers where labor force participation is currently lowest. Accordingly, the effects of an (additional) educational expansion take time to materialize. A scenario for a broad educational expansion, moving 25 percent of persons in each educational group to the next-higher educational level, adds slightly more than 1 million workers to the U.S. labor force in 2060 (compared to our baseline, which already includes a rise in education levels). This effect is stronger when we extend the projection horizon, adding about 3.3 million workers to the U.S. labor force in 2080 (compared to our baseline).

Our projections highlight that improvements in the labor market integration of people with health limitations provide a particularly promising avenue to increase labor force participation and thus cushion the negative economic effects of demographic aging. If the health gap in participation rates in the U.S. were similar to Sweden’s, the labor force in 2060 would be stronger by about 13.4 million persons than in our baseline projections. This result has to be interpreted as an upper bound, as differences in population health may result in greater difficulties or even the impossibility to achieve participation rates in the United States that are as high as those observed in Sweden. This notwithstanding, our estimates indicate that measures that prolong working lives by promoting health as well as by improving the labor market inclusion of workers with temporary or permanent health impairments can make substantial contributions to mitigate the impact of demographic ageing on the labor market and the economy.

The microsimulations based on microWELT for Germany and other European countries illustrated in Horvath et al. (2021) show that the effects of an expansion of education and improved population health affect employment and hours worked more than labor force participation. The effects are larger because low educational attainment and health limitations increase the risk of unemployment and of underemployment, compared to higher education and good health. Thus, most what-if-scenarios lead to a greater increase in the number of employed persons and the number of hours worked than in the number of labor force participants.

Own calculations based on CPS data for the U.S. and EU-SILC data for the European countries.

## Data Availability

This research is based on public comparative microdata which are accessible for the purpose of scientific analysis from the respective sources. Users are not allowed to pass on the data to third parties. The data sources for all calculations, tables and graphs are given in the article. Simulation results are based on statistically estimated or calculated parameters using these data. The model documentation is available under microWELT.eu.

## Appendix 1

See Figs. 13, 14, Tables 5, 6, 7, 8, and 9.

**Table 5:** Odds ratios from logistic regression for LFP, United States

	Young (age 15–24)		Prime age (25 to 54)				Retirement (55 to 74)	
	Male	Female	Male	Female		Female with kids	Male	Female
Currently in education (Base category: no)								
Yes	0.15***	0.24***						
	(0.000)	(0.000)						
Highest level of education (base category: below highschool)								
Highschool	2.00***	2.21***	1.43***	1.82***		1.91***	1.4296 ***	1.8218 ***
	(0.003)	(0.004)	(0.001)	(0.002)		(0.002)	(0.001)	(0.002)
College	2.43***	2.77***	1.70***	2.78***		2.83***	1.6757 ***	2.609 ***
	(0.004)	(0.004)	(0.002)	(0.003)		(0.003)	(0.002)	(0.002)
University	3.04***	4.59***	2.80***	3.65***		3.77***	2.7767 ***	3.3475 ***
	(0.008)	(0.012)	(0.003)	(0.005)		(0.003)	(0.003)	(0.003)
Health status (base category: good)								
Bad			0.21***	0.37***		0.64***	0.2346 ***	0.4616 ***
			(0.000)	(0.000)		(0.000)	(0.000)	(0.000)
Age of youngest child in family (base category: none or at least 18 years of age)								
0–2						0.43***		
						(0.000)		
3–5						0.49***		
						(0.000)		
6–10						0.78***		
						(0.001)		
10–17						0.98***		
						(0.001)		
Constant	1.77***	1.31***	2.65***	0.79***		0.70***	5.8976 ***	1.6124 ***
	(0.002)	(0.002)	(0.002)	(0.002)		(0.002)	(0.012)	(0.003)

*p* < 0.05; ***p* < 0.01; ****p* < 0.001. Logistic regressions also include 5-year age indicators for young and prime age groups and single-year age indicators for the retirement age group (not displayed).

**Table 6:** Odds ratios from logistic regression for LFP, Germany

	Young (age 15–24)		Prime age (25 to 54)			Retirement (55 to 74)	
	Male	Female	Male	Female	Female with kids	Male	Female
Currently in education (base category: no)							
Yes	0.13***	0.23***					
	(0.001)	(0.001)					
Highest level of education (base category: ISCED 2 or lower)							
ISCED 3	3.99***	4.90***	1.53***	1.57***	1.78***	1.3405***	1.7348***
	(0.01)	(0.013)	(0.003)	(0.003)	(0.006)	(0.003)	(0.004)
ISCED 4	4.38***	6.15***	2.03***	2.39***	2.48***	2.0738***	2.3807***
	(0.034)	(0.12)	(0.005)	(0.008)	(0.008)	(0.008)	(0.006)
ISCED 5+	1.48***	5.97***	2.29***	3.57***	2.94***	2.1538***	3.4936***
	(0.009)	(0.004)	(0.005)	(0.008)	(0.001)	(0.006)	(0.009)
Health status (base category: good)							
Bad			0.30***	0.34***	0.66***	0.3275***	0.3364***
			(0.000)	(0.000)	(0.002)	(0.001)	(0.001)
Age of youngest child in family (base category: none or at least 18 years of age)							
0–2					0.23***		
					(0.001)		
3–5					0.54***		
					(0.002)		
6–10					0.73***		
					(0.002)		
10–17					0.98***		
					(0.001)		
Constant	1.72***	0.71***	0.09***	1.16***	5.16***	10.2678***	5.1027***
	(0.006)	(0.002)	(0.000)	(0.002)	(0.025)	(0.012)	(0.003)

*p* < 0.05; ***p* < 0.01; ****p* < 0.001. Logistic regressions also include 5-year age indicators for young and prime age groups and single-year age indicators for the retirement age group (not displayed).

**Table 7:** Labor force participation rates by gender and age, United States 2020 and 2060

	Female		Male		Total	
	2020	2060	2020	2060	2020	2060
15–19	25.2	24.8	29.9	29.6	33.0	32.3
20–24	64.2	63.9	63.5	63.4	66.2	64.2
25–29	79.4	81.0	86.6	86.5	82.6	83.5
30–34	80.5	81.8	92.7	92.6	83.1	84.3
35–39	81.5	82.5	93.9	94.5	82.8	84.1
40–44	85.8	87.8	94.3	94.6	82.7	84.3
45–49	87.0	89.2	93.2	93.7	81.8	83.5
50–54	84.9	88.8	92.1	92.5	79.2	80.8
55–59	78.3	82.8	88.2	89.0	70.7	73.2
60–64	55.3	60.8	64.4	65.9	59.6	62.0
65–69	13.8	22.2	20.2	26.1	32.8	37.7
Total	64.4	64.6	73.0	70.7	66.6	67.1

Labor force participation rates based on microWELT simulations.

**Table 8:** Labor force participation rates by gender and age, Germany 2020 and 2060

	Female		Male		Total	
	2020	2060	2020	2060	2020	2060
15–19	34.6	33.8	31.4	30.7	27.7	27.2
20–24	64.3	62.6	68.0	65.7	63.8	63.6
25–29	77.6	78.7	87.4	88.3	83.1	83.7
30–34	75.5	77.3	90.6	91.3	86.7	87.2
35–39	75.5	77.3	90.1	90.9	87.8	88.4
40–44	75.8	77.9	89.6	90.5	90.1	91.2
45–49	76.7	78.7	87.2	88.1	90.1	91.4
50–54	74.3	76.2	84.2	85.3	88.5	90.6
55–59	66.2	69.3	75.4	77.1	83.3	85.9
60–64	54.8	57.8	64.8	66.2	59.8	63.4
65–69	28.9	34.5	37.3	41.0	16.8	24.1
Total	61.7	62.9	71.6	71.4	68.7	67.6

Labor force participation rates based on microWELT simulations.

**Table 9:** Odds ratios for education transitions by parents’ highest level of education

	United States		Germany	
	Female	Male	Female	Male
Odds for transition from education level 1 to 2				
Parents education: medium	6.8	4.9	5.8	4.6
Parents education: high	24.2	8.9	17.3	8.3
Odds for transition from education level 2 to 3				
Parents education: medium	3.3	3.2	3.1	2.5
Parents education: high	11.9	9.6	9.2	8.8
Odds for transition from education level 3 to 4				
Parents education: medium	5.6	3.3	2.2	1.9
Parents education: high	19.4	11.0	7.5	7.5

Own calculations based on CPS Annual Social and Economic Supplements 2017, OECD PIAAC data and EU-SILC 2014. Parents’ education is grouped into three categories. “Low” (reference category): less than highschool (ISCED levels 0–2 for Germany), “medium”: high school diploma/some college but no degree (ISCED levels 3 and 4 for Germany) and high: college degree or higher (ISCED level 5 + for Germany).

## Footnotes

1 These projections, however, do not yet take into account the effects of the COVID-19 pandemic.

2 From 65.4 percent in 2000 to 76.7 percent in 2019. This figure refers to the age group 15 to 64 years, see Destatis, https://www.destatis.de/DE/Themen/Arbeit/Arbeitsmarkt/Erwerbstaetigkeit/Tabellen/erwerbstaetigenquoten-gebietsstand-geschlecht-altergruppe-mikrozensus.html.

3 Predicted probabilities, ${\widehat{p}}_{i}$, are calculated by$\widehat{p}=\mathrm{exp}({\widehat{X}}^{^{\prime}}(\beta ))/(1+\mathrm{exp}({\widehat{X}}^{^{\prime}}(\beta ))$.

4 However, Laun and Palme (2020) find that the rise in labor force participation in Sweden has coincided with improvements in health, educational attainment, and stricter disability insurance screening. Coile (2018) concludes that the decline of health insurance for U.S. retirees led some workers with no health insurance other than through their job to work until they qualify for Medicare, thus contributing to longer working lives.

5 The projections also differ by the definition of age groups, the level of disaggregation, and the granularity of published results. For instance, the projections provided by the CBO as supplement to its 2021 Long-term Budget Outlook define working age people as those aged between 16 and 89 years (CBO, 2021).

6 Unlike in the case of the education scenarios, a separate presentation of cohort-specific and total effects is not meaningful here.
